# Simultaneous SNP identification and assessment of allele-specific bias from ChIP-seq data

**DOI:** 10.1186/1471-2156-13-46

**Published:** 2012-09-05

**Authors:** Yunyun Ni, Amelia Weber Hall, Anna Battenhouse, Vishwanath R Iyer

**Affiliations:** 1 Center for Systems and Synthetic Biology, Institute for Cellular and Molecular Biology, Section of Molecular Genetics and Microbiology, University of Texas at Austin, Austin, TX, 78712, USA

**Keywords:** SNPs, Transcription factors, ChIP-seq, Genotyping, Allele-specific

## Abstract

**Background:**

Single nucleotide polymorphisms (SNPs) have been associated with many aspects of human development and disease, and many non-coding SNPs associated with disease risk are presumed to affect gene regulation. We have previously shown that SNPs within transcription factor binding sites can affect transcription factor binding in an allele-specific and heritable manner. However, such analysis has relied on prior whole-genome genotypes provided by large external projects such as HapMap and the 1000 Genomes Project. This requirement limits the study of allele-specific effects of SNPs in primary patient samples from diseases of interest, where complete genotypes are not readily available.

**Results:**

In this study, we show that we are able to identify SNPs de novo and accurately from ChIP-seq data generated in the ENCODE Project. Our de novo identified SNPs from ChIP-seq data are highly concordant with published genotypes. Independent experimental verification of more than 100 sites estimates our false discovery rate at less than 5%. Analysis of transcription factor binding at de novo identified SNPs revealed widespread heritable allele-specific binding, confirming previous observations. SNPs identified from ChIP-seq datasets were significantly enriched for disease-associated variants, and we identified dozens of allele-specific binding events in non-coding regions that could distinguish between disease and normal haplotypes.

**Conclusions:**

Our approach combines SNP discovery, genotyping and allele-specific analysis, but is selectively focused on functional regulatory elements occupied by transcription factors or epigenetic marks, and will therefore be valuable for identifying the functional regulatory consequences of non-coding SNPs in primary disease samples.

## Background

Single nucleotide polymorphisms (SNPs) have been associated with normal variation in biological traits as well as many human diseases [1,2]. With recent advances in genotyping and sequencing technologies, the number of known SNPs has increased dramatically. Several international collaborative projects such as the HapMap project [3] and the 1000 Genomes Project [4] have genotyped millions of SNPs in hundreds of human individuals. In the dbSNP 129 build (http://www.ncbi.nlm.nih.gov/projects/SNP/), approximately 52 million SNPs are cataloged in humans. However, despite the availability of these massive datasets, our understanding of the functional importance of most genomic variants is still very limited. Genome-wide association (GWA) studies have been the main method to associate genomic variants with a variety of phenotypes including diseases. SNP genotyping is performed in large numbers of disease individuals and controls to find statistically significant associations between SNPs and diseases [5]. So far, GWA studies have identified approximately 7000 SNPs or regions as disease associated [1], although in most cases neither the causal SNPs nor the molecular mechanisms are known (http://www.genome.gov/gwastudies/).

Although SNPs located within coding regions that affect protein sequences are the most obvious candidates for functional SNPs, among the 7000 disease associated SNPs, only about 7% affect coding regions. For the majority of SNPs occurring in non-coding regions and shown to be associated with disease risk, there are two possibilities for explaining this association. First the identified or imputed SNPs could directly contribute to disease risk. Alternatively, it has been proposed that identified SNPs could be in linkage disequilibrium (LD) with rarer and unknown causative SNPs that are not directly assayed or imputed [6]‐[8]. In either case, it raises the question of how common or rare variants in non-coding regions could contribute to disease risk. One explanation for the functional effects of non-coding SNPs in introns and intergenic regions is that they affect transcription regulation, splicing or other aspects of RNA processing or stability [9,10]. For example, a SNP upstream of MUC5B has been shown to regulate its expression and is associated with familial interstitial pneumonia and idiopathic pulmonary fibrosis [11]. Disease-associated SNPs have also been reported to directly affect transcription factor binding. Two non-coding regulatory SNPs in FGFR2 affect binding of the transcription factors Oct-1/Runx2 and C/EBPβ, leading to an increased expression of FGFR2 in the rarer homozygous genotypes which have increased breast cancer risk [12]. A SNP upstream of MYC has been shown to affect the binding of transcription factor YY1 which may serve to regulate MYC expression in prostate cancers [13]. Two SNPs in the intronic promoter of MDM2, an oncogene that downregulates the tumor suppressor TP53, have independent and opposite effects on the binding of the transcription factor SP1. However, the combination of both SNPs into a commonly observed haplotype reduces SP1 binding in the MDM2 promoter and reduces breast and ovarian cancer risk, likely by reducing MDM2 expression [14,15]. While these studies shed light on the functional basis of regulatory SNPs, they account for a very small fraction of known disease associated non-coding SNPs.

We have previously shown that the transcription factor CTCF exhibits allele-specific binding bias at hundreds of heterozygous SNP sites in the human genome [16]. This study and others [17] showed that not only is there widespread allele-specific binding of transcription factors, but suggested a genetic basis for this specificity, with nucleotide polymorphisms directly affecting the binding of transcription factors. In principle therefore, it should be possible to examine transcription factor binding and other features of gene expression such as chromatin modifications and RNA expression levels in disease cells to identify whether polymorphisms associated with disease risk indeed affect transcription factor binding, chromatin, and/or RNA in an allele-specific manner. However, most previous analyses of allele-specific transcription factor binding and chromatin depended upon the availability of individual genotypes from the 1000 Genomes Project, and therefore cannot be easily performed in primary patient samples, where whole-genome genotyping data based on deep sequencing is generally not available. We have now developed an approach for simultaneous SNP discovery and transcription factor allele-specific binding analysis from ChIP-seq data we have generated in the ENCODE Project, without reference to preexisting genotyping information. This approach has enabled us to examine allele specific transcription factor binding in any cell where ChIP-seq data is available and paves the way for extending such analysis of other sequencing-based readouts of gene regulatory mechanisms to primary cell samples from individuals without pre-existing genotype data.

## Results

### SNP discovery from ChIP-seq data

We carried out SNP discovery from ChIP-seq data that we generated for the transcription factor CTCF in 10 human cell lines, including six lymphoblastoid cell lines that had been previously sequenced and genotyped by the 1000 Genomes Project, embryonic stem cells (H1 ESCs), vascular endothelial cells (HUVEC), and normal and disease fibroblasts (Table 1) [16,18]. In addition we carried out SNP discovery from RNA pol II (RNAPII) and H3K4me3 ChIP-seq data from GM12878 cells, an ENCODE Tier 1 cell line. Besides the above call sets which we analyzed in detail, we also called SNPs in H3K4me3 and/or H3K27me3 ChIP-seq data generated by ENCODE from 17 other cell lines as well as from RNA-seq data in GM12891 to demonstrate that the pipeline is broadly applicable (Methods). In order to identify SNPs, we adapted the variant discovery software suite developed by the 1000 Genomes Project, the Genome Analysis ToolKit (GATK) [19,20]. Raw Illumina sequences were aligned to the NCBI36 (hg18) reference human genome sequence with BWA [21] and the alignment was first processed to minimize the effect of duplicated reads, sequencing and alignment errors on genotype calls. The cleaned up alignments were then used to calculate genotype likelihoods and initial SNP calls were made (Methods, Additional file 1: Figure S1). Because of our interest in examining how polymorphisms affect the binding of a sequence-specific transcription factor, we focused exclusively on single nucleotide substitutions and did not consider small insertions/deletions (indels) or larger structural variants in our analysis. While small indels were readily observed in the ChIP-seq alignments (see below), larger structural variants would be missed given the nature of the ChIP-seq data. We filtered the initial SNP call set using two methods, model based filtering and hard filtering. We carried out model-based filtering as implemented in GATK. Briefly, a Gaussian mixture model was built with those initially called SNPs that overlapped with known high-quality SNPs identified by the HapMap and the 1000 Genomes Projects, using their specified annotations. Then all called SNPs were fitted into the model and thresholds were set so that 99% of the known high-quality SNPs were retained [19,20]. Hard filtering was a heuristic approach in which we excluded SNPs from the initial called set that met the following criteria: more than 2 SNPs within 10 base pairs, more than 10% of the sequence reads overlapping the SNP were not uniquely aligned, SNPs with quality scores less than 50, SNPs overlapping with repeat regions [22], SNPs within 5 bp of indels and SNPs located within regions with exceptionally high read coverage [23]. We considered only SNPs that passed both the model filter and the hard filter for all subsequent analysis presented here.

**Table 1:** Statistics of SNP discovery

Cell line	factor	aligned reads	Ht5 regions	total SNPs	novel SNPs	Ti/Tv
GM12878	CTCF	30,400,254	0.40%	112,999	2989(2.6%)	2.04
GM12891	CTCF	28,282,066	0.45%	137,056	3443(2.5%)	2.18
GM12892	CTCF	41,857,998	0.75%	206,349	5439(2.6%)	2.13
GM19238	CTCF	31,125,372	0.53%	154,211	7474(4.8%)	2.11
GM19239	CTCF	24,857,361	0.41%	153,092	7034(4.6%)	2.13
GM19240	CTCF	32,009,059	0.45%	144,088	7832(5.4%)	2.11
GM12878	RNAPII	85,763,827	0.55%	152,071	5720(3.8%)	2.03
GM12878	H3K4me3	74,464,458	1.95%	200,675	8231(4.1%)	1.99
FB8470	CTCF	39,394,358	0.47%	173,336	4344(2.5%)	2.11
H1 ESC	CTCF	14,462,504	0.28%	94,398	2308(2.4%)	2.18
Progeria	CTCF	46,925,953	0.47%	220,871	5328(2.4%)	2.06
HUVEC	CTCF	21,734,605	0.32%	55,492	1117(2.0%)	2.08

GM lines are lymphoblastoid cells resequenced by the 1000 Genomes Project. FB8470 is a normal fibroblast line and Progeria refers to a fibroblast line obtained from patients with the disease. H1 ESCs are human embryonic stem cells and HUVECs are human vascular endothelial cells. Ht5 regions are regions covered by at least 5 reads in ChIP-seq data. The fraction of the genome represented in Ht5 regions is shown. Novel SNPs are with reference to dbSNP129. Ti/Tv is the transition to transversion ratio.

Table 1 shows a summary of the identified SNPs. Among all CTCF ChIP samples, the total number of SNPs discovered was proportional to the number of aligned reads, with an average of about 150,000 SNPs per individual. Since different transcription factors have different binding properties, the same number of reads may lead to different genome coverage. For example, RNAPII ChIP-seq in GM12878 had more aligned reads than H3K4me3 ChIP-seq, but the fraction of the genome covered by more than 5 reads in RNAPII ChIP-seq (0.55%) was less than that in H3K4me3 ChIP-seq. As a result, more SNPs were discovered in H3K4me3 ChIP-seq than in RNAPII. The transition to transversion ratio (Ti/Tv) for de novo identified SNPs was around 2.1, close to what is estimated in mammals [19,24].

### SNPs discovered from ChIP-seq overlap well with 1000 genomes project SNPs

In the pilot phase of the 1000 Genomes Project, two trios with both parents and a child (CEU and YRI) were sequenced to an average depth of 42X coverage, and complete genotype information for these six individuals was published [4]. To test whether the SNPs we discovered from our ChIP-seq data from these samples were in agreement with the published genotypes, we performed overlap analysis between SNPs discovered from CTCF ChIP-seq and those published by the 1000 Genomes Project (Pilot 2 SNPs, July, 2010 release).

More than 90% of the SNPs we identified de novo using CTCF ChIP-seq data in the previously resequenced cell lines overlapped Pilot 2 SNP calls. Because DNA recovered after ChIP represents only a small portion of the genome, we discovered only 3-7% of all Pilot 2 SNPs. However, if we considered only Pilot 2 SNPs within regions where we had at least 5X coverage in the CTCF ChIP, more than 70% of such Pilot 2 SNPs were recovered in our discovery (Table 2). Furthermore, the percent recovery of Pilot 2 SNPs in ChIP-ed regions correlated well with the proportion of the genome covered in ChIP-ed regions (Additional file 1: Figure S2). SNPs we discovered from ChIPseq datasets for RNAPII and H3K4me3 in GM12878 also showed the same high overlap with Pilot 2 SNPs in this cell line (Table 2).

**Table 2:** Quality assessment of the discovered SNPs. Pilot 2 SNPs are identified by the 1000 Genomes Project

cell line	factor	total SNPs	overlap with Pilot 2	Pilot 2 SNPs recovered by ChlP	Pilot 2 SNPs in Ht5 region	Pilot 2 SNPs in Ht5 regions recovered by ChlP	validated/tested G1000 SNPs	Validated/Tested novel SNPs
GM12878	CTCF	112,999	94.14%	3.85%	11,908	75.37%	15/17	4/5
GM12891	CTCF	137,056	90.39%	4.55%	12,837	74.12%	13/13	4/4
GM12892	CTCF	206,349	92.23%	6.95%	20,772	76.54%	13/13	7/7
GM19238	CTCF	154,211	92.25%	4.47%	18,863	74.50%	11/11	3/3
GM19239	CTCF	153,092	94.33%	4.43%	14,844	70.37%	15/15	2/2
GM19240	CTCF	144,088	94.48%	4.08%	17,143	74.09%	10/10	3/3
GM12878	RNAPII	152,071	93.45%	5.14%	8,368	77.84%	5/6	2/2
GM12878	H3K4me3	200,675	93.19%	6.76%	44,755	79.40%	1/1	1/1
FB8470	CTCF	173,336	N/A	N/A	N/A	N/A	N/A	0/0
H1 ESC	CTCF	94,398	N/A	N/A	N/A	N/A	N/A	0/0
Progeria	CTCF	220,871	N/A	N/A	N/A	N/A	N/A	7/7
HUVEC	CTCF	55,492	N/A	N/A	N/A	N/A	N/A	7/7
Progeria	RNAPII	150,157	N/A	N/A	N/A	N/A	N/A	5/7

G1000 SNPs are ChIP-seq discovered SNPs that overlap with Pilot 2 SNPs. N/A is not applicable, as these cells were not genotyped by the 1000 Genomes Project.

To independently verify SNPs found in ChIP-seq data, we carried out Sanger sequencing of genomic DNA at several randomly selected loci. We examined a total of 133 sites across 8 cell lines. All tested sites except for six were confirmed to be correctly identified SNPs, suggesting a 5% error rate for our ChIP-seq genotypes (Table 2, Additional file 1: Figure S3). However, 2 of these 6 discrepancies were cases where the genotype called by ChIP-seq matched the 1000 Genomes genotype, and in the remaining cases, either the alleles identified by ChIP-seq matched the alleles known to occur at that location, or the discrepancy could be explained by low sequencing coverage by ChIP-seq, suggesting that our error rate is in fact lower (Additional file 1: Table S1).

The overlap between ChIP and Pilot 2 SNPs described above consider a SNP as overlapping between the two sets when their location and the called alt allele was the same. If we required an exact genotype match between the two sets, the percent overlap was somewhat lower (Additional file 1: Figure S4A). ChIP SNPs had a lower heterozygosity rate than Pilot 2 SNPs, suggesting that some heterozygous SNPs had insufficient read coverage and therefore were called homozygous (Additional file 1: Figure S4B). If we filtered out SNPs with low read coverage, the heterozygosity rate of ChIP SNPs was similar to that of Pilot 2 SNPs, and the exact genotype overlap between ChIP SNPs and Pilot 2 SNPs was also restored to about 90% (Additional file 1: Figure S4C). We were also able to identify small indels from ChIP-seq data and the majority of these indels also overlapped with those called by the 1000 Genomes Project (Additional file 1: Table S2).

### SNPs overlapping with 1000 genomes project and novel SNPs are qualitatively similar

**Figure 1:**
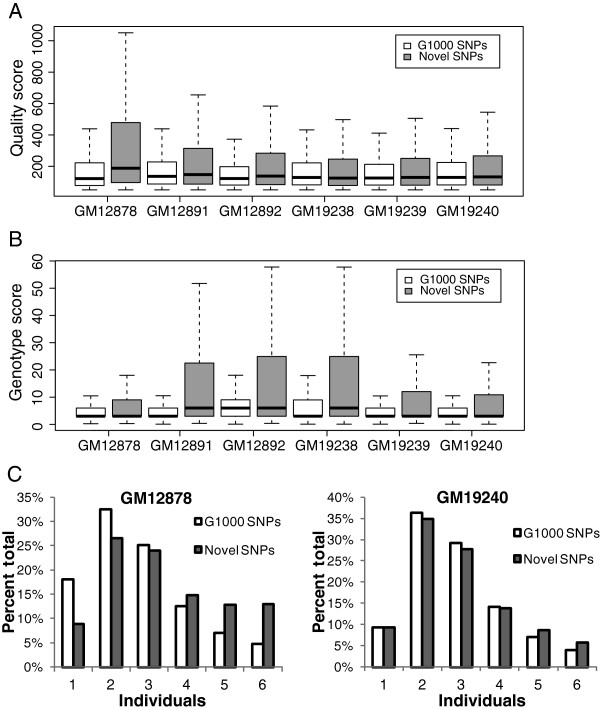
**G1000 SNPs and novel SNPs are qualitatively similar.** (**A**) SNP quality scores and (**B**) genotype quality scores of the G1000 SNPs and novel SNPs are shown for each of the indicated cell lines as standard box-plots. G1000 SNPs are those identified de novo from ChIP-seq data but overlap with the 1000 Genomes Project Pilot 2 SNP calls. (**C**) Individual distribution of SNPs. G1000 SNPs and novel SNPs discovered in GM12878 and GM19240 CTCF samples were categorized according to their individual distribution. For example, ‘1’ represents SNPs found in only one of the six individuals, ‘2’ represents SNPs found in two individuals, etc.

In order to characterize in more detail the SNPs we discovered de novo from ChIP-seq data, we separated them into SNPs that overlapped with those found by the 1000 Genomes Project Pilot 2 dataset (referred to here as G1000 SNPs) and those that were not found in Pilot 2 and were therefore novel (novel SNPs). First, we established by genomic sequencing that our novel SNPs were as accurate as G1000 SNPs (Table 2, Additional file 1: Figure S3). We next investigated whether G1000 SNPs were different from novel SNPs in terms of their quality metrics. SNP quality scores reported by the GATK pipeline are Phred-scaled probability scores of the existence of a polymorphism. We found that the median quality scores of novel SNPs were equal to or greater than the mean quality scores of G1000 SNPs (Figure 1A). We then compared the genotype scores which represent the confidence of the genotype called between G1000 SNPs and novel SNPs, and found that novel SNPs had equal or higher genotype scores than G1000 SNPs (Figure 1B). We also examined the distribution of SNPs among six individuals. If novel SNPs were largely erroneous, they might be expected to be found in a single individual but not shared across multiple individuals. However, more than half of the novel SNPs we found were observed in at least 3 individuals and the overall distribution across individuals was also indistinguishable between G1000 and novel SNPs (Figure 1 C, Additional file 1: Figure S5). Finally, we checked our novel SNPs against the larger set of SNP calls made by the 1000 Genomes project at low coverage across a large number of individuals from the same populations. 71% - 86% of the SNPs that were novel in a given cell type were indeed found in other individuals in the low coverage set, suggesting that they were not spurious (Additional file 1: Table S3).

### Genomic and chromosomal distribution of discovered SNPs

**Figure 2:**
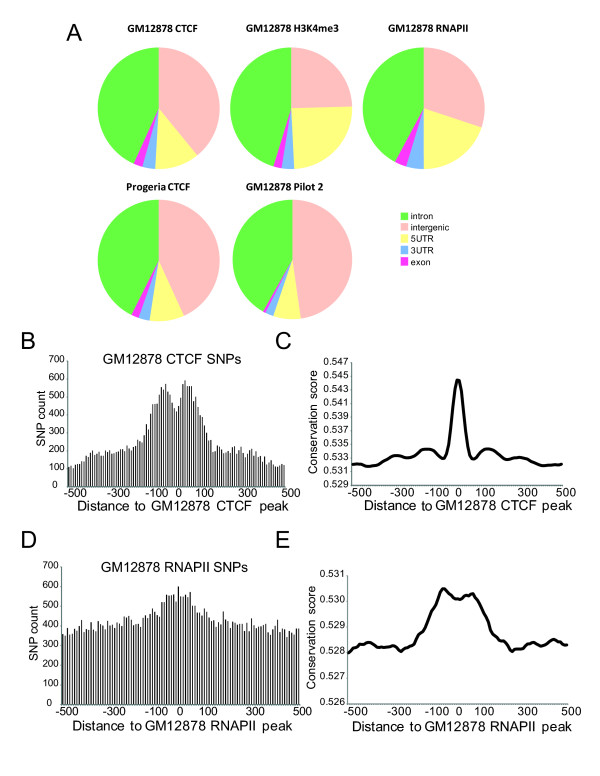
**Properties of discovered SNPs.** (**A**) SNP distribution relative to annotated genes. CTCF, H3K4me3 and RNAPII refer to SNPs discovered from ChIP-seq data. (**B**) Distribution of GM12878 CTCF SNPs around ChIP peaks. 10 bp bins were generated from the highest points of peaks and the number of SNPs was counted in each bin. SNP counts were added up across all peaks in every bin and plotted. (**C**) Distribution of conservation scores around GM12878 CTCF peaks. Conservation scores were assigned to each of the 10 bp bins and the average was plotted. (**D**) Distribution of GM12878 RNAPII SNPs and (**E**) phastCons conservation scores around GM12878 RNAPII ChIP peaks,

We examined the genomic distribution of the SNPs we identified from ChIP-seq data. Compared with Pilot 2 SNPs, a larger proportion of the SNPs we discovered from RNAPII and H3K4me3 ChIP-seq data were localized to the 5’ UTR, reflecting RNAPII and H3K4me3 localization over the transcription start site (TSS) region (Figure 2A). In contrast, SNPs from CTCF ChIP-seq data showed a genomic location profile similar to Pilot 2 SNPs, both in the GM12878 lymphoblastoid cell line and in a Progeria fibroblast line (Figure 2A), reflecting the distinct binding distribution of CTCF.

When SNP discovery is carried out using ChIP-seq data, it might be expected that the highest SNP density occurs around significant ChIP peaks which are identified on the basis of normalized read coverage. We therefore investigated the distance between identified SNPs and the corresponding ChIP peak positions. Most SNPs from CTCF ChIP-seq data were indeed localized around −200 to +200 bp from CTCF peak binding positions, but interestingly, the number of CTCF SNPs dropped at the very center of CTCF peaks (Figure 2B). Since CTCF is evolutionarily conserved and its binding sites are also believed to be well conserved [25], we tested whether CTCF binding peaks were more highly conserved than the surrounding regions. When phastCons conservation scores from 28 vertebrate species [26] were profiled around CTCF peaks, a sharp increase in conservation was observed at CTCF binding centers (Figure 2 C). The lower density of de novo SNPs that we observed at the centers of CTCF binding peaks is therefore likely due to increased evolutionary constraint at CTCF binding sites. We also observed a similar drop in SNP density of 1000 Genomes Project SNPs in at the center of CTCF binding sites (Additional file 1: Figure S6A).

In contrast to CTCF SNPs, SNPs discovered from RNAPII ChIP-seq data showed only a modest enrichment around RNAPII binding peaks (Figure 2D). Phylogenetic conversation was higher over a broader range around RNAPII peak centers (Figure 2E) where the Pilot 2 SNP density was also lower (Additional file 1: Figure S6B). This lower overall SNP density around RNAPII binding sites as well as at TSS (Additional file 1: Figure S6C) may minimize any potential increase in the discovery of SNPs at RNAPII binding sites from RNAPII ChIP-seq data.

**Figure 3:**
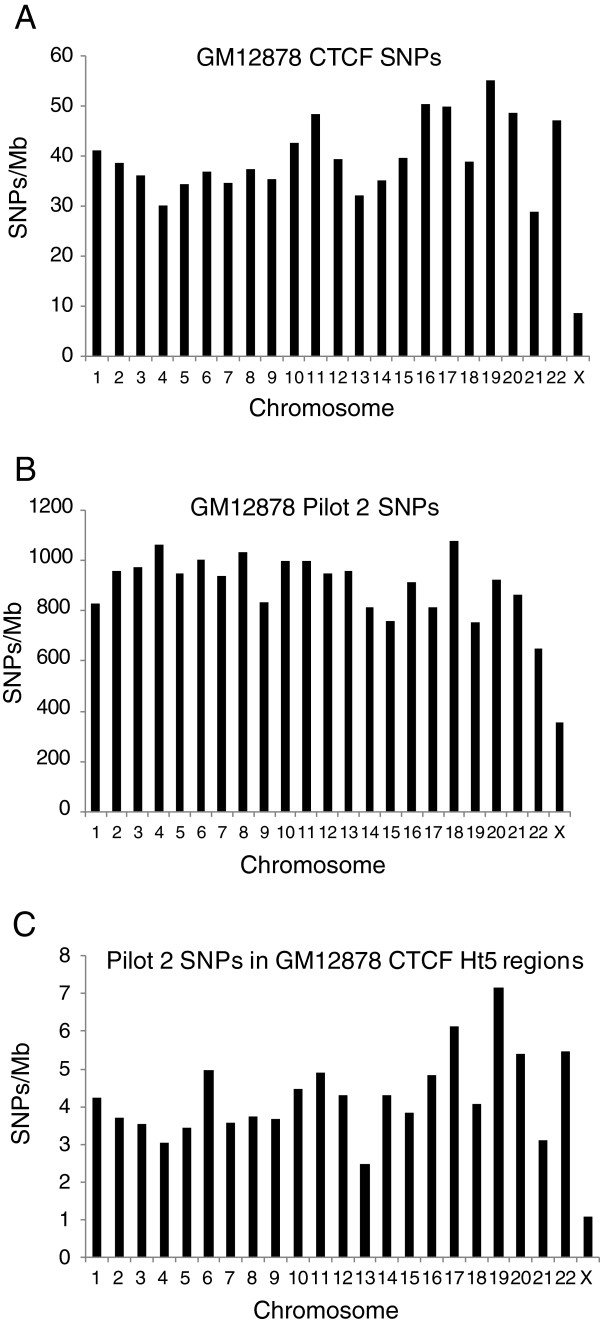
**Chromosomal distribution of SNPs.** Chromosomal distribution of SNPs is plotted as SNPs/Mb. Chromosome number is shown on the X axis. Data is shown for (**A**) GM12878 CTCF SNPs (**B**) GM12878 Pilot 2 SNPs and (**C**) Pilot 2 SNPs within GM12878 CTCF ChIP Ht5 regions, which are regions with at least 5X coverage in ChIP-seq data.

Analysis of the chromosomal distribution of SNPs discovered from CTCF ChIP-seq data showed that the relative SNP density on the X chromosome was markedly lower than that of the autosomes (Figure 3A). This was not a peculiarity of our SNP discovery pipeline as applied to ChIP-seq data, because when we analyzed Pilot 2 SNPs, we saw that the X chromosome similarly had a lower SNP density than autosomes (Figure 3B). The more pronounced bias for the X chromosome within SNPs discovered from ChIP-seq data (compare Figures 3A and 3B) is not a SNP calling artifact because when we calculated the density of Pilot 2 SNPs within CTCF binding sites, the relative X chromosome SNP density was likewise lower than that of autosomes (Figure 3 C).

### SNP discovery from ChIP-seq data is proportional to sequencing depth

**Figure 4:**
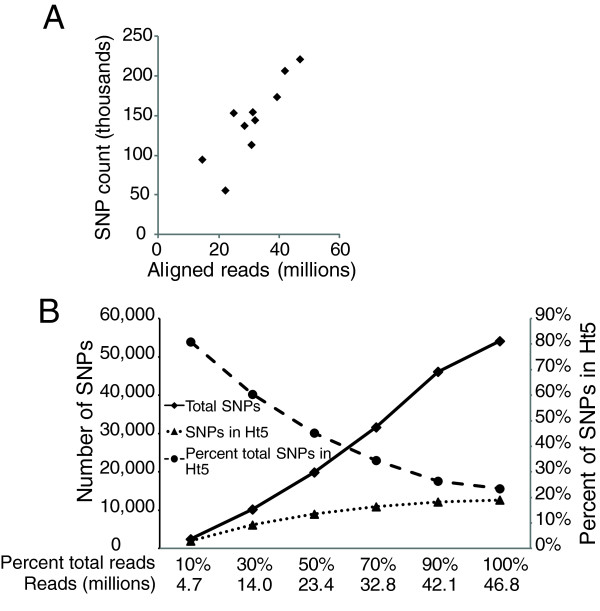
**Read depth and SNP discovery.** (**A**) Correlation between total number of discovery SNPs and number of aligned reads. The number of discovered SNPs from CTCF ChIP-seq in 10 cell lines was plotted against the number of aligned reads in each cell line. (**B**) Effect of increasing read depth on SNP discovery. A random sample as indicated on the X axis was taken from Progeria CTCF ChIP-seq reads and SNP discovery was performed using this subset of reads. The number of SNPs discovered in each sample as well as number of SNPs discovered in ChIP-ed regions (with at least 5X read coverage) is plotted. ChIP-ed regions were defined with the full set of reads (100% of the total reads).

From SNP discovery with CTCF ChIP-seq data in multiple cell lines, we found that the number of SNPs discovered varied considerably between cell lines (Table 1). Because the sequencing depth, that is, the total amount of sequence generated, differed between cell lines, we asked whether the difference in sequencing depths contributes to the difference in number of SNPs discovered. We found that the number of discovered SNPs indeed correlated well with the number of aligned reads (Figure 4A).

Since SNPs within transcription factor binding regions are of the greatest interest from the perspective of understanding how genetic variation affects transcription factor binding, we were interested in understanding how increased sequencing depth affected SNP discovery in those ChIP-ed regions. For this purpose, we used CTCF ChIP-seq data in Progeria fibroblasts where we had the greatest sequencing depth. We defined ChIP-ed regions as regions covered by 5 or more reads and analyzed the total number of SNPs, and SNPs discovered within ChIP-ed regions as a function of sequencing depth. While the total number of discovered SNPs increased linearly with increasing sequencing depth, the number of SNPs within ChIP-ed regions increased more slowly and almost plateaued after ~32 million reads. Furthermore, with increased sequencing depth, the percent of total SNPs within ChIP-ed regions decreased, suggesting that the additional SNPs discovered with deeper sequencing occur outside of the ChIP-ed regions and are therefore less likely to be relevant for transcription factor binding (Figure 4B).

### Consistent allele-specific binding bias at SNPs discovered from ChIP-seq data

We have previously found that at a subset of assayable transcription factor binding sites, CTCF displays an allelic binding bias in that there is significant difference in its occupancy of the two alleles [16]. This allele-specific binding was consistent across individuals and appeared to be heritable. Our ability to accurately identify SNPs de novo from ChIP-seq data in principle enables us to detect allele-specific transcription factor binding bias even in the absence of prior genotype information. To confirm this, we first performed allele-specific binding bias analysis at SNP sites discovered de novo from CTCF ChIP-seq data in the six lymphoblastoid cell lines (Methods).

**Figure 5:**
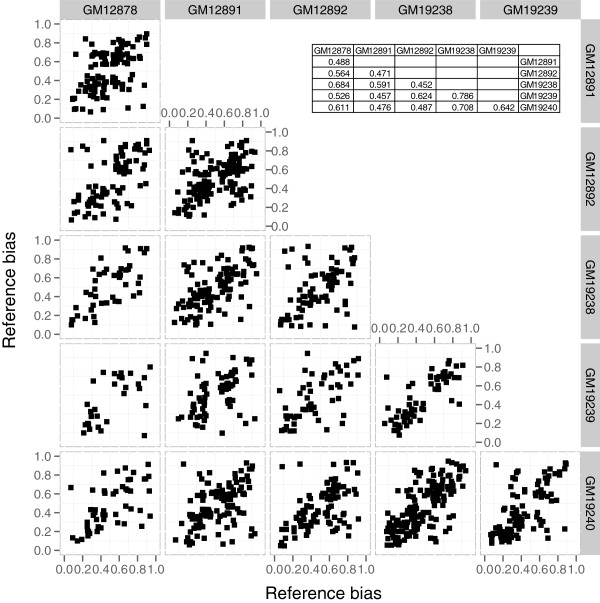
**Bias analysis with discovery SNPs.** Each plot is a scatter plot of percent reference allele (reads with reference allele/total reads) at shared heterozygous SNPs between two individuals as indicated on the top and right. SNPs that were significantly biased in at least one of the two individuals with an FDR corrected P-value less than 0.05 are included. The inset tables show the Spearman correlation of the bias between two individuals (top) and the Spearman P-values (bottom).

A pairwise comparison of binding bias between two individuals, at shared heterozygous SNPs where the bias was statistically significant in at least one of the two individuals, showed that binding biases were largely concordant across individuals (Figure 5). The Spearman correlation coefficients of the binding biases between individuals were positive and highly significant in every case (Figure 5 inset tables). In particular, the bias values for most SNPs were located in the upper right or lower left quadrants, indicating that the direction of the binding bias was consistent between individuals. These results with SNPs we discovered de novo from ChIP-seq data recapitulate the findings from previous analysis which used then available genotypes released by the 1000 Genomes Project, and were mirrored in a similar analysis we performed using a more recent set of genotyped SNPs from the 1000 Genomes Project Pilot 2 release (Additional file 1: Figure S7). The set of significantly biased de novo discovered SNPs included in Figure 5 and the set of significantly biased Pilot 2 SNPs also overlap well (Additional file 1: Table S4).

We then extended this bias analysis to normal and disease fibroblasts that had not been previously genotyped, and found CTCF allelic binding bias between these two lines was similarly well correlated (Additional file 1: Figure S8). Thus, de novo SNP discovery using ChIP-seq data allows the measurement of allele-specific binding in the absence of any genotyping information and confirms the genetic basis of such allele-specific phenomena in primary and disease cells.

### SNPs found in ChIP-seq data are informative for non-coding disease-associated variants

**Figure 6:**
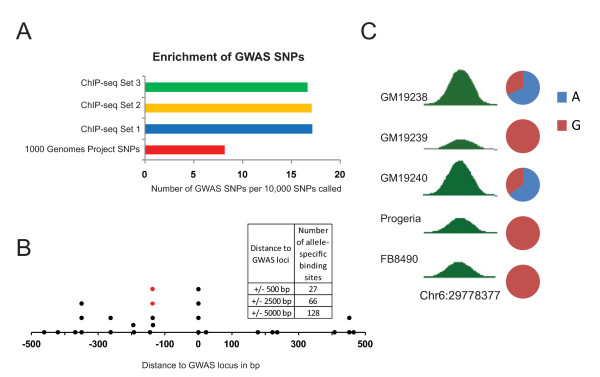
**SNPs discovered from ChIP-seq and disease-associated SNPs.** (**A**) The proportions (per 10,000) of SNPs that were identified either by the 1000 Genomes Project Pilot 2 set (1000 Genomes trio) or discovered from ChIP-seq data in this study (ChIP-seq), that were also associated with diseases based on the NHGRI GWAS catalog (www.genome.gov/gwastudies) are shown. ChIP-seq Set 1 refers to those SNPs discovered de novo from ChIP-seq data that were also called by the 1000 Genomes Pilot 2, to allow direct comparison. ChIP-seq Set 2 refers to SNPs called from ChIP-seq data generated by us in cell lines that were not genotyped by the 1000 Genomes Project. ChIP-seq Set 3 refers to SNPs called from ChIP-seq data from other ENCODE labs in cell lines that were not genotyped by the 1000 Genomes Project. (**B**) Allele-specific binding near GWAS SNPs. Each point represents a significantly biased allele-specific binding event of CTCF at the indicated distance from a GWAS locus. Shown here are all allele-specific binding loci within +/− 500 bp of a GWAS site (which would be at position 0). Multiple points at the same distance represent allele-specific binding in different individuals. (**C**) An example from the set of allele-specific binding sites near a GWAS SNP. The two sites indicated by red dots in 'B' correspond to the heterozygous sites in 'C'. The read density of ChIP-seq data at the binding site is shown in selected individuals. When heterozygous, the pie chart indicates relative occupancy of the two alleles.

To assess the biological utility of SNPs discovered directly from ChIP-seq data, we examined their overlap with SNPs that have been associated with human diseases through GWA studies [1]. Only 8 out of every 10,000 SNPs that were identified in the 1000 Genomes Pilot 2 Project were associated with a disease, but this proportion more than doubled when we looked at the ChIP-seq SNPs we identified. 17 out of every 10,000 SNPs that we found de novo by ChIP-seq overlapped with a disease associated SNP (Figure 6A). By restricting this overlap analysis to those de novo ChIP-seq SNPs that were also found in Pilot 2, we determined that this enrichment for disease SNPs within the ChIP-seq dataset is highly significant (P = 8.2 x 10-99 by hypergeometric probability distribution). We found that a similarly higher proportion of ChIP-seq SNPs that we discovered across other cell lines that had not been genotyped by the 1000 Genomes project overlapped with disease-associated SNPs (Figure 6A).

Allele-specific binding biases could potentially reveal differences between normal and disease risk alleles, especially at non-coding SNPs. We therefore analyzed if any of the significantly biased allele-specific binding sites that we identified after genotyping and allele-specific analysis occurred near disease-associated SNPs identified by GWAS. We identified dozens of cases of significant allele-specific binding at distances ranging from 500 bp to 5 kb from SNPs associated with various diseases (Figure 6B, Additional file 1: Table S5). Since this size range is well below the median haplotype block size of about 45 kb in humans [3,27,28], a majority of these allele-specific binding sites we have identified are likely to occur on the same haplotype block as the GWAS SNP. An example of such a SNP is shown in Figure 6 C. In cells (GM19238 and GM19240) where the loci are heterozygous, the A allele is preferentially bound by CTCF. Consistent with this, in cells that have two G alleles at this location (GM19239, FB8460 and Progeria), CTCF binding is weaker than at the heterozygous sites. When they occur on the appropriate haplotypes, such allele-specific binding in non-coding regions could effectively represent a functional distinction between the nearby normal and risk alleles.

## Discussion

### SNP discovery and transcription factor binding allelic bias analysis from ChIPseq data

We developed an approach to simultaneously discover SNPs and perform transcription factor binding bias analysis using ChIP-seq data. Over 90% of our discovered SNPs in six human lymphoblastoid cell lines overlap with the SNPs identified by the 1000 Genomes Project (Table 1). A similar proportion of SNPs that we discovered but were missed by the 1000 Genomes Project were in fact accurate as checked by Sanger sequencing. From the point of view of using such discovered SNPs to assess allele-specific binding events, the small number of cases where a heterozygous site is misidentified as homozygous do not constitute a problem since they are effectively "false negative" sites which would not be assayable for allele-specificity. Most discovered SNPs were close to transcription factor binding sites and the distribution of SNPs mimicked that of ChIP peaks (Figure 2). Due to the selectivity of ChIP, only a small fraction of all known SNPs were discovered; however, the majority of SNPs within ChIP-ed regions and therefore at transcription factor binding sites were recovered (Table 1). Furthermore, although increasing read depth leads to more SNPs, most SNPs in ChIP-ed regions were discovered with 20–30 million reads, which is a typical ChIP-seq dataset, suggesting that this type of analysis does not require very deep sequencing (Figure 4B).

The difference in SNP density on the X chromosome relative to autosomes is interesting. Many factors can lead to a difference in diversity between the X chromosome and autosomes, such as different effective population size, male mutation bias and natural selection [29,30]. We speculate that variants in functional regions on the X chromosome, for example, at transcription factor binding sites, are subject to a stronger natural selection bias than similar mutations on autosomes owing to the hemizygosity of the X chromosome. The difference in SNP density between the X chromosome and autosomes is therefore larger in SNPs discovered from CTCF ChIP-seq data, which are focused on functional regions, than in 1000 Genomes Project SNPs, which are largely intergenic.

### Utility of SNP genotyping from ChIP-seq and related data

Since many disease-associated polymorphisms are in non-coding regions and are believed to affect gene regulation, a subset of such variants could affect transcription factor binding, chromatin structure or RNA processing. Determining the allelic effects of disease-associated SNPs on transcription factor binding, histone modifications, DNA methylation or DNaseI hypersensitivity would therefore be a powerful approach to directly assess the functional regulatory impact of non-coding polymorphisms. While it is of greatest interest to perform such studies on patient samples, one limitation is that complete genotypes are rarely available for primary patient samples. Our approach overcomes this limitation by enabling genotyping (SNP calling) and assessment of allele-specific effects directly from the ChIP or other functional experimental data, without requiring a separate genotyping dataset. We found that SNPs at transcription factor binding sites discovered from ChIP-seq data show an overrepresentation of disease-associated SNPs. Interestingly, previous studies have also shown an enrichment of GWAS SNPs in active intergenic chromatin regions that are defined partly by high CTCF binding [31]. Additionally, we found that many allele-specific binding events occur near non-coding disease-associated variants identified by GWAS. Such allele-specific binding potentially represents a functional distinction between the normal and disease alleles, which is currently not known for the majority of non-coding disease variants identified by GWAS. We readily identified the examples shown in Figure 6 and Additional file 1: Table S5 by analysis of a small number of ChIP-seq datasets. Similar analysis as we describe here, applied to the systematic datasets of genome-wide transcription factor binding, chromatin status, and gene expression will undoubtedly identify such distinctions for many more disease-associated loci, which can then be tested in patient samples.

While genotyping using arrays or deep sequencing might appear to be straightforward, arrays generally cover only a small subset of all possible polymorphisms, and whole genome resequencing requires far deeper sequencing coverage than required for typical ChIP-seq or RNA-seq assays. The fact that we could identify and verify SNPs that were missed in the 1000 Genomes Project genotypes suggests that the selective focus of our method on biochemically functional elements offers an advantage even over whole-genome sequencing, likely because our sequencing coverage over these loci is higher at these enriched loci. While it is possible that a subset of phenotypic effects are due to rare "private" variants, distinguishing whether a rare non-coding variant has a functional consequence or is neutral is challenging. The approach outlined here can not only identify such rare variants but also immediately establish whether it significantly affects the experimental assay that was used to identify it in the first place. We propose that by analogy to exome sequencing which is aimed at identifying variants in coding regions, the approach we have used here can be considered to be "targetome" sequencing, in which variants in functional non-coding regions are identified along with information on how these variants affect enrichment in the functional assay. Despite its potential, technical issues are likely to arise in certain domains. For example, one application might be to evaluate the allelic effects of non-coding somatic mutations and germline polymorphisms in cancer, a disease where the underlying affected cells are readily identifiable (as tumors) and can be isolated for experimental analysis. However, cancer cells are often aneuploid and tumor samples from patients are often genetically heterogeneous, so bias analysis for transcription factor binding or chromatin must account for the background allele composition. SNP arrays have been used to determine copy number differences in cancer cells [32], so in parallel to ChIP-seq analysis as outlined here, it should be possible to determine the copy number of each allele in the input genomic DNA from the same sample, and use this quantitative information as the background while calculating the significance of any allele-specific binding bias.

Although our study focused on ChIP-seq data, combining ChIP-seq and RNA-seq allele-specific analysis in the same samples will clarify the relationship between non-coding regulatory polymorphisms and allele-specific gene expression. Since transcription factor and chromatin changes are likely some distance away from the transcribed region along the linear chromosome, such an understanding will also require knowledge of SNP phasing along individual chromosomes, but haplotype inference based on statistical approaches or direct haplotype resolved sequencing methods can fill this gap [33,34]. Allele-specific events of all types can thus be measured in primary and disease cells and will shed light on how genotypic variation corresponds to variation in phenotype, including diseases.

## Conclusions

Our approach demonstrates that it is possible to discover SNPs with very high accuracy and simultaneously perform transcription factor binding bias analysis directly from ChIP-seq data. While the overwhelming majority of SNPs discovered from ChIP-seq data coincide with published genotype data, the small number of novel SNPs that we found appear to be indistinguishable from previously published variants based on several criteria. Using SNPs discovered de novo from ChIP-seq data, we confirmed and extended previous observations that allele-specific biases tend to occur towards the same allele across individuals and are therefore consistent with an underlying genetic basis for these binding differences. SNPs discovered from ChIP-seq data are strongly enriched for disease-associated variants, and allele-specific binding at many of these SNPs could allow a functional distinction to be made between normal and disease haplotypes.

## Methods

### Data source

All CTCF and RNAPII ChIP-seq and peak finding was performed by our group as described earlier [18] and the data is available from the ENCODE project web site (http://genome.ucsc.edu/ENCODE/). H3K4me3 and H3K27me3 ChIP-seq data was also downloaded from the ENCODE website. RNA-seq data for GM12891 was from a previously published study [35]. The statistics of these data sets are in Table 1 and Additional file 1: Table S6. 1000 Genome Project SNP calls were as in their Pilot 2 release [4]. The NHGRI GWAS catalog (http://www.genome.gov/gwastudies) was downloaded and the data as accessed on Feb 23 2012 was used for our analysis.

### SNP calling with GATK

Raw ChIP-seq sequencing reads were aligned to the human reference genome NCBI36 (hg18) with BWA [21]. To ensure optimal alignment, we used the reference genome provided with GATK that contained all 24 chromosome sequences as well as additional contigs that have not been assembled. GATK was then used to mark duplicated reads, realign around insertions/deletions and recalibrate quality scores for the alignments. The Unified Genotyper was used to generate initial SNP calls in vcf format which was then further filtered. The initial SNP calls were evaluated with a Gaussian Mixture Model (GATK Variant Recalibrator) and outliers were discarded. After this initial filtering, the SNPs in the following categories were then filtered out: more than 2 SNPs within 10 base pairs, more than 10% overlapping reads are not uniquely mapped, SNPs with quality scores less than 50, SNPs overlapping with repeat regions [22], SNPs within 5 bp of insertions/deletions, SNPs located within regions with exceptionally high read coverage [23].

### Allelic binding bias analysis

Bias analysis was performed as described previously [16]. To avoid alignment biases favoring reads containing the reference allele, we constructed individual-specific reference genomes that contained the SNPs discovered in that individual from the ChIP-seq data, representing both the reference and the alternate allele in the case of heterozygous SNPs. We then carried out a second round of alignments to these individual-specific genomes. We counted the number of reads covering each allele at heterozygous SNPs and expressed the ChIP-seq binding bias as the proportion of reads that contain the reference allele. We calculated the significance of this bias using a binomial model, assuming under the null hypothesis that both alleles of a heterozygous SNP would be equally represented in the ChIP DNA. P-values were corrected for multiple testing using the false discovery rate (FDR) on the set of SNPs where the difference of allele counts between the two alleles was at least 6. The lists of significantly biased binding sites from de novo identified SNPs as well as from 1000 Genomes Project Pilot 2 SNPs are provided as Additional file 2: Table S7 and Additional file 3: Table S8 respectively.

## Competing interests

The authors declare no competing interests.

## Authors’ contributions

VRI and YN conceived of and designed the study. YN and AB carried out the computational analysis. AWH performed the experimental validation. VRI and YN wrote the manuscript. All authors read and approved the final manuscript.

## Supplementary Material

Additional file 1: **Figure S1.** Diagram of SNP discovery pipeline. **Figure S2.** Numbers of Pilot 2 SNPs rediscovered correlate with ChIP-seq coverage. For each trio cell line, the percentage of Pilot 2 SNPs rediscovered with ChIP-seq data is plotted together with percent of the genome with at least 5X coverage from ChIP-seq. **Figure S3.** Validation of de novo discovered SNPs by genomic sequencing. The top row shows examples of SNPs discovered de novo from ChIP-seq data that were also genotyped in that individual by the 1000 Genomes Pilot 2 Project. The remainder are examples of SNPs discovered de novo from ChIP-seq data but missed in the 1000 Genomes Pilot 2 set in that individual (GM cell lines) or found in ungenotyped lines (HUVEC, Progeria). The top of each panel shows the genomic DNA sequence, with the SNP at the center in bold. Chromosomal coordinates, transcription factor/histone modification, and cell line are listed below the chromatogram. **Figure S4.** SNP calling in low coverage regions. (A) Location overlap and genotype overlap between CTCF ChIP-seq SNPs and Pilot 2 SNPs. Location overlap is when the SNP location and alleles match, but sometimes only one allele of a heterozygous genotype is observed in the other set. Genotype overlap refers to an exact genotype match. (B) Percent heterozygosity for CTCF ChIP-seq discovery SNPs and Pilot 2 SNPs. (C) Read number filtering increases discovery SNP heterozygosity and genotype overlap with Pilot 2 SNPs. SNPs covered by less than the indicated number of reads were filtered out. Blue bars represent the number of SNPs passing the filter. Red squares represent SNP heterozygosity and green triangles represent the percent genotype overlap with Pilot 2 SNPs, both on the secondary Y axis on the right. **Figure S5.** Individual distribution of SNPs. G1000 SNPs and novel SNPs discovered in the indicated GM cell lines. CTCF ChIP-seq samples were categorized according to their individual distribution. ‘1’ represents SNPs found in only one of the six individuals, ‘2’ represents SNPs found in two people and so on. **Figure S6.** Pilot 2 SNP distribution around (A) CTCF and (B) RNAPII ChIP peak centers and (C) transcription start sites. (D) Conservation scores around transcription start sites. All distances are in bp. **Figure S7.** CTCF allelic binding bias at Pilot 2 SNPs was plotted similarly as in Fig. 5. The inset tables show the Spearman correlation coefficients (top) and Spearman *P* values (bottom). **Figure S8.** CTCF allelic binding bias at discovered SNPs in Progeria and FB8470 (normal) fibroblast cells. **Table S1.** Description of apparent errors. This table lists all 6 discrepancies that we observed between genotypes called from ChIP-seq data and our genomic Sanger sequencing validation (127 out of 133 were exactly correct). For errors 1 and 3, the ChIP-seq data recovered the alternate allele and called it homozygous, but the reference allele was apparently not observed at sufficient coverage. Errors 2 and 4 are discrepant between the ChIP-seq and Sanger genotyping, but our ChIP-seq call matched the 1000 Genomes Pilot 2 genotype. For errors 5 and 6, the ChIP-seq data called it heterozygous and Sanger sequencing reported homozygous (similar to errors 2and 4), but the two alleles reported by ChIP-seq correspond to the two alleles known to occur at that position (in other individuals) according to dbSNP 129. **Table S2.** Indels called from ChIP-seq data overlap with 1000 Genomes Project indel calls. **Table S3.** Novel SNPs found by ChIP-seq overlap with SNPs found in other individuals in the same population in the 1000 Genomes Project low coverage data. **Table S4.** Overlap between biased (that is, allele-specific) SNPs discovered from ChIP-seq data and biased Pilot 2 SNPs.**Table S5.** Significantly biased allele-specific CTCF binding sites within 500 bp of a GWAS SNP locus. *P*-val refers to the significance of the allele-specificity binding bias at a heterozygous SNP.** Table S6.** SNP calling from H3K4me3 and/or H3K27me3 ChIP-seq data in 17 additional cell lines (ENCODE data) as well as from RNA-seq data in GM12891 (from Toung *et al*., Genome Res. (2011) 21:991-8). Click here for file

Additional file 2 CTCF allele-specific binding at SNPs discovered de novo from CTCF ChIP-seq. SNPs with an FDR corrected bias *P* value of less than 0.05 are included. Each tab contains information for one individual. Click here for file

Additional file 3 CTCF allele-specific binding at Pilot 2 SNPs. SNPs with an FDR corrected bias *P* value of less than 0.05 are included. Each tab contains information for one individual. Click here for file
