# Supplementary material for: Simultaneous SNP identification and assessment of allele-specific bias from ChIP-seq data
Source: BMC Genet. 2012 Sep 5;13:46. doi: 10.1186/1471-2156-13-46 (PMC3434080; doi:10.1186/1471-2156-13-46)
Supplement: Additional file 1: — Figure S1. Diagram of SNP discovery pipeline. Figure S2. Numbers of Pilot 2 SNPs rediscovered correlate with ChIP-seq coverage. For each trio cell line, the percentage of Pilot 2 SNPs rediscovered with ChIP-seq data is plotted together with percent of the genome with at least 5X coverage from ChIP-seq. Figure S3. Validation of de novo discovered SNPs by genomic sequencing. The top row shows examples of SNPs discovered de novo from ChIP-seq data that were also genotyped in that individual by the 1000 Genomes Pilot 2 Project. The remainder are examples of SNPs discovered de novo from ChIP-seq data but missed in the 1000 Genomes Pilot 2 set in that individual (GM cell lines) or found in ungenotyped lines (HUVEC, Progeria). The top of each panel shows the genomic DNA sequence, with the SNP at the center in bold. Chromosomal coordinates, transcription factor/histone modification, and cell line are listed below the chromatogram. Figure S4. SNP calling in low coverage regions. (A) Location overlap and genotype overlap between CTCF ChIP-seq SNPs and Pilot 2 SNPs. Location overlap is when the SNP location and alleles match, but sometimes only one allele of a heterozygous genotype is observed in the other set. Genotype overlap refers to an exact genotype match. (B) Percent heterozygosity for CTCF ChIP-seq discovery SNPs and Pilot 2 SNPs. (C) Read number filtering increases discovery SNP heterozygosity and genotype overlap with Pilot 2 SNPs. SNPs covered by less than the indicated number of reads were filtered out. Blue bars represent the number of SNPs passing the filter. Red squares represent SNP heterozygosity and green triangles represent the percent genotype overlap with Pilot 2 SNPs, both on the secondary Y axis on the right. Figure S5. Individual distribution of SNPs. G1000 SNPs and novel SNPs discovered in the indicated GM cell lines. CTCF ChIP-seq samples were categorized according to their individual distribution. ‘1’ represents SNPs found in only one of the six indi [file 1471-2156-13-46-S1.pdf]

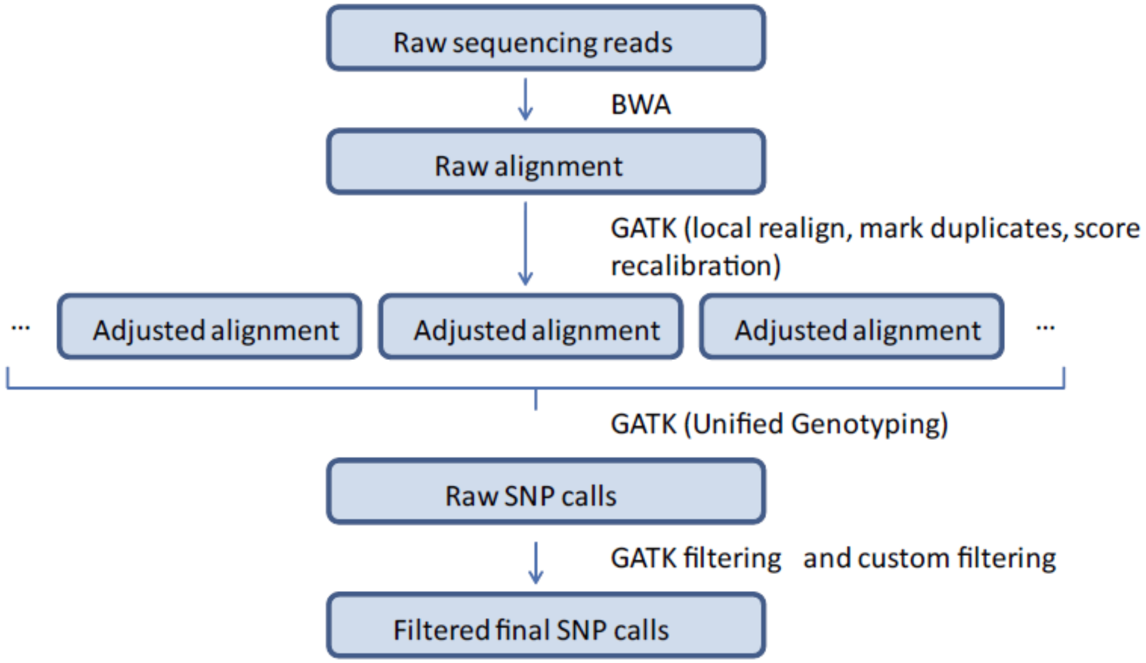

Supplemental Figure S1. Diagram of SNP discovery pipeline.

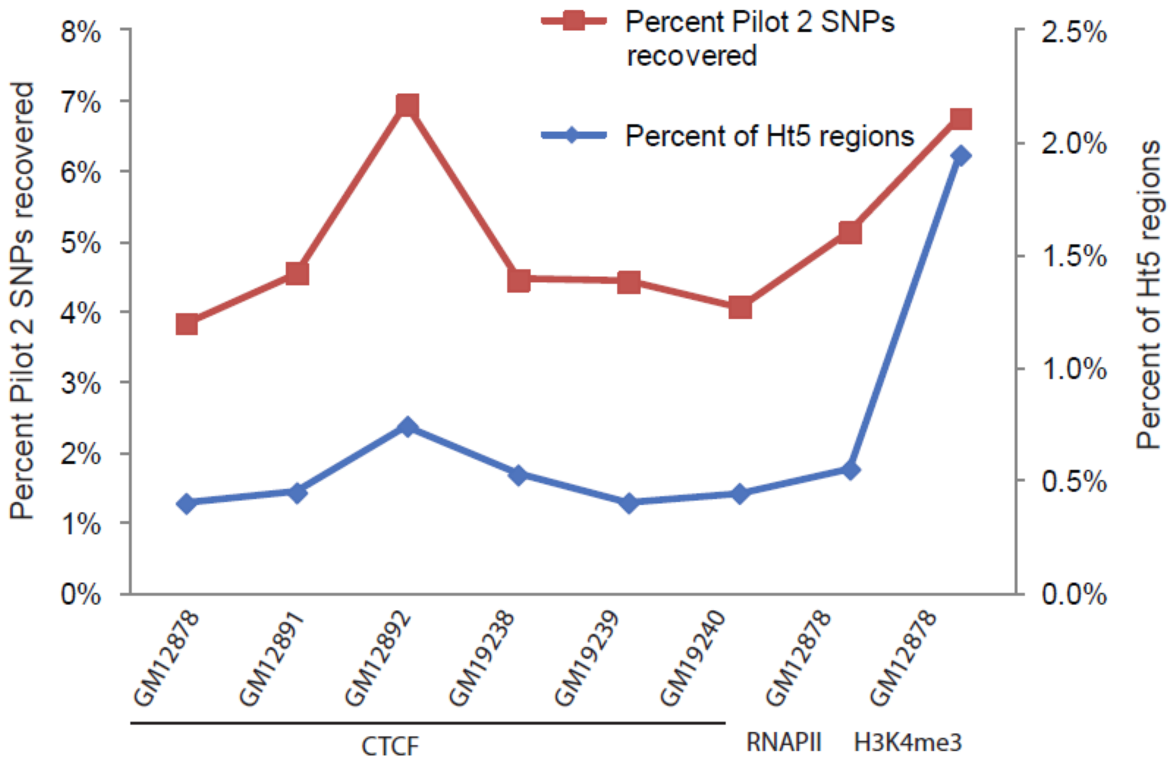

Supplemental Figure S2. Numbers of Pilot 2 SNPs rediscovered correlate with ChIP-seq coverage. For each trio cell line, the percentage of Pilot 2 SNPs rediscovered with ChIP-seq data is plotted together with percent of the genome with at least 5X coverage from ChIP-seq.

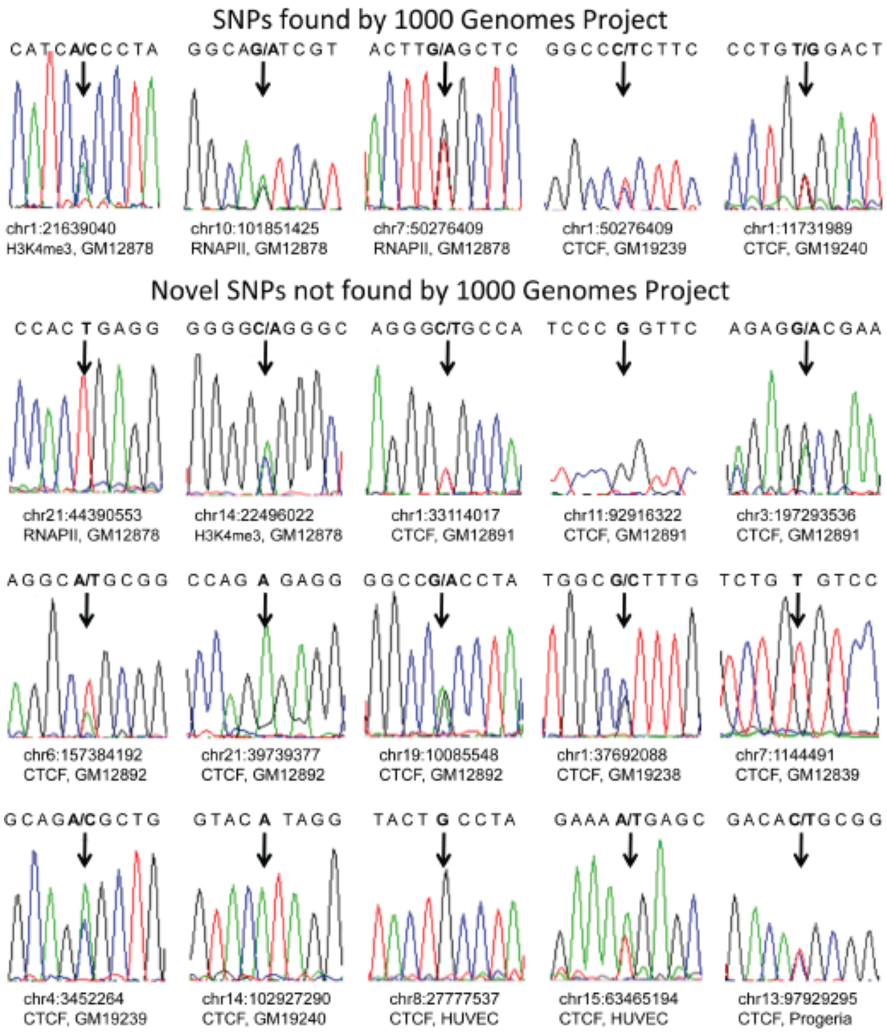

Supplemental Figure S3. Validation of de novo discovered SNPs by genomic sequencing. The top row shows examples of SNPs discovered de novo from ChIP-seq data that were also genotyped in that individual by the 1000 Genomes Pilot 2 Project. The remainder are examples of SNPs discovered de novo from ChIP-seq data but missed in the 1000 Genomes Pilot 2 set in that individual (GM cell lines) or found in un-genotyped lines (HUVEC, Progeria). The top of each panel shows the genomic DNA sequence, with the SNP at the center in bold. Chromosomal coordinates, transcription factor/histone modification, and cell line are listed below the chromatogram.

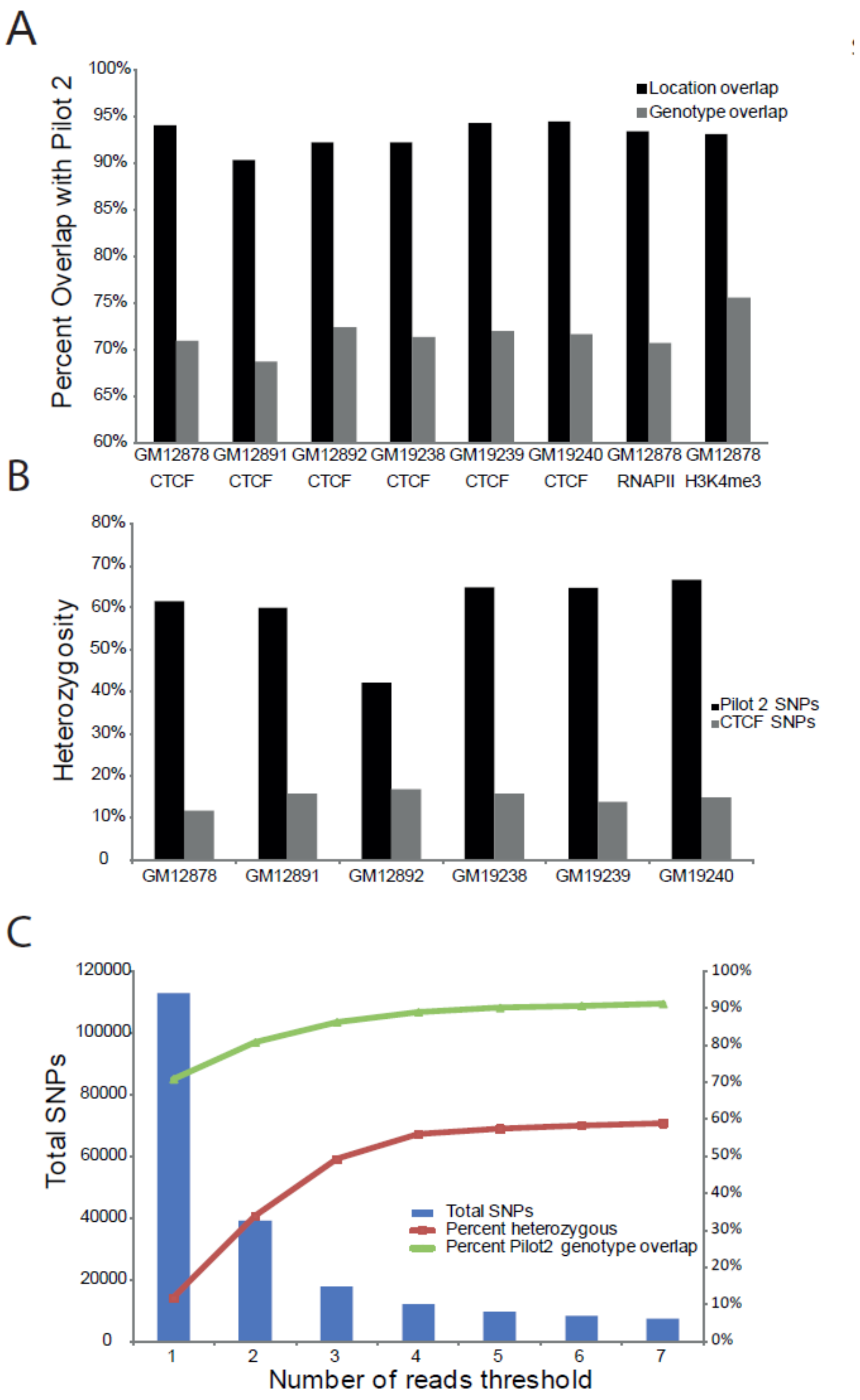
A
100%
95%
90%
85%
80%
75%
70%
65%
60%
Percent Overlap with Pilot 2
Location overlap
Genotype overlap
GM12878 CTCF
GM12891 CTCF
GM12892 CTCF
GM19238 CTCF
GM19239 CTCF
GM19240 CTCF
GM12878 RNAPII
GM12878 H3K4me3
B
80%
70%
60%
50%
40%
30%
20%
10%
0
Heterozygosity
Pilot 2 SNPs
CTCF SNPs
GM12878
GM12891
GM12892
GM19238
GM19239
GM19240
C
120000
100000
80000
60000
40000
20000
0
Total SNPs
100%
90%
80%
70%
60%
50%
40%
30%
20%
10%
0%
Total SNPs
Percent heterozygous
Percent Pilot2 genotype overlap
1
2
3
4
5
6
7
Number of reads threshold

Supplemental Figure S4. SNP calling in low coverage regions. (A) Location overlap and genotype overlap between CTCF ChIP-seq SNPs and Pilot 2 SNPs. Location overlap is when the SNP location and alleles match, but sometimes only one allele of a heterozygous genotype is observed in the other set. Genotype overlap refers to an exact genotype match. (B) Percent heterozygosity for CTCF ChIP-seq discovery SNPs and Pilot 2 SNPs. (C) Read number filtering increases discovery SNP heterozygosity and genotype overlap with Pilot 2 SNPs. SNPs covered by less than the indicated number of reads were filtered out. Blue bars represent the number of SNPs passing the filter. Red squares represent SNP heterozygosity and green triangles represent the percent genotype overlap with Pilot 2 SNPs, both on the secondary Y axis on the right.

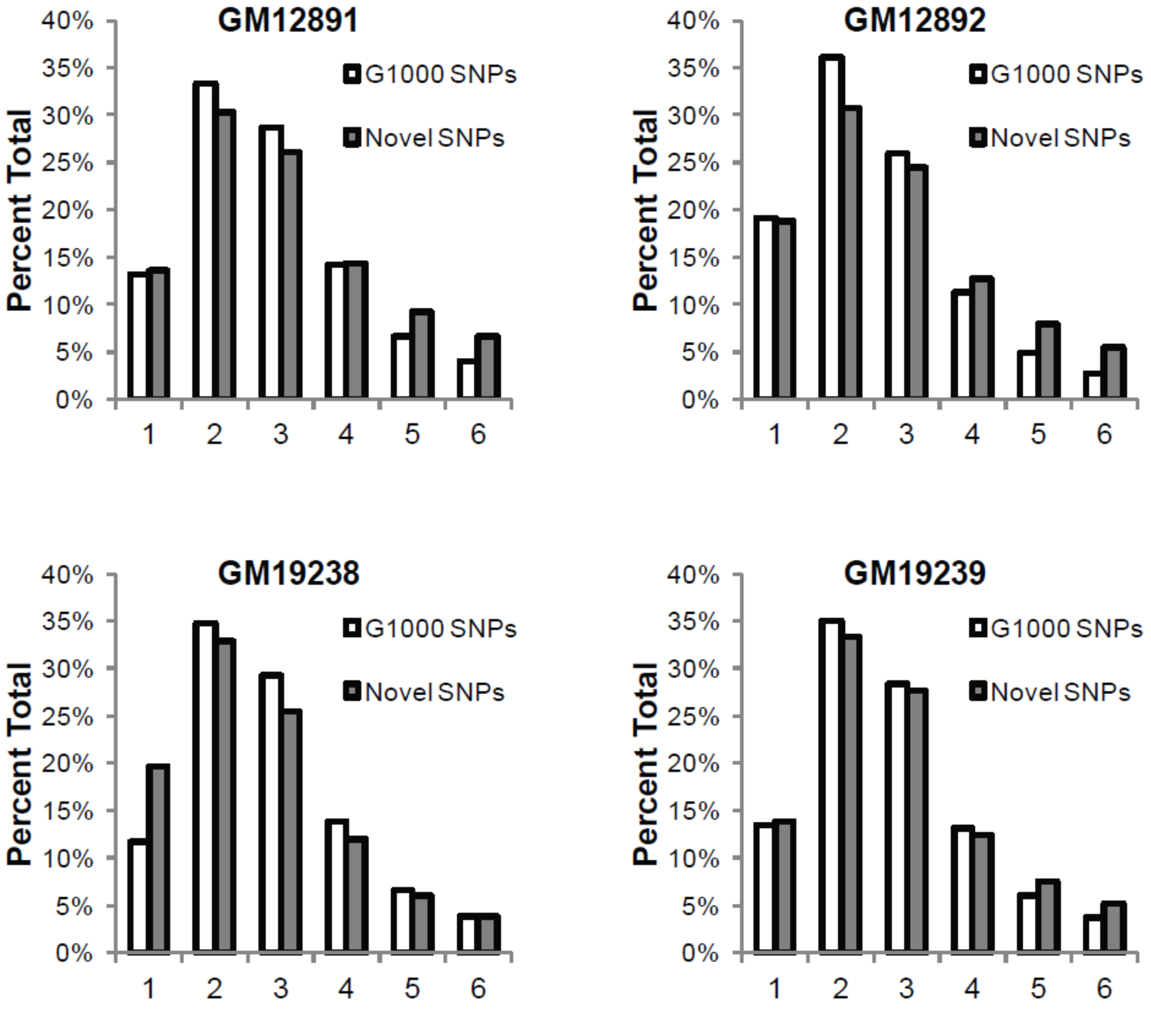

Supplemental Figure S5. Individual distribution of SNPs. G1000 SNPs and novel SNPs discovered in the indicated GM cell lines. CTCF ChIP-seq samples were categorized according to their individual distribution. ‘1’ represents SNPs found in only one of the six individuals, ‘2’ represents SNPs found in two people and so on.

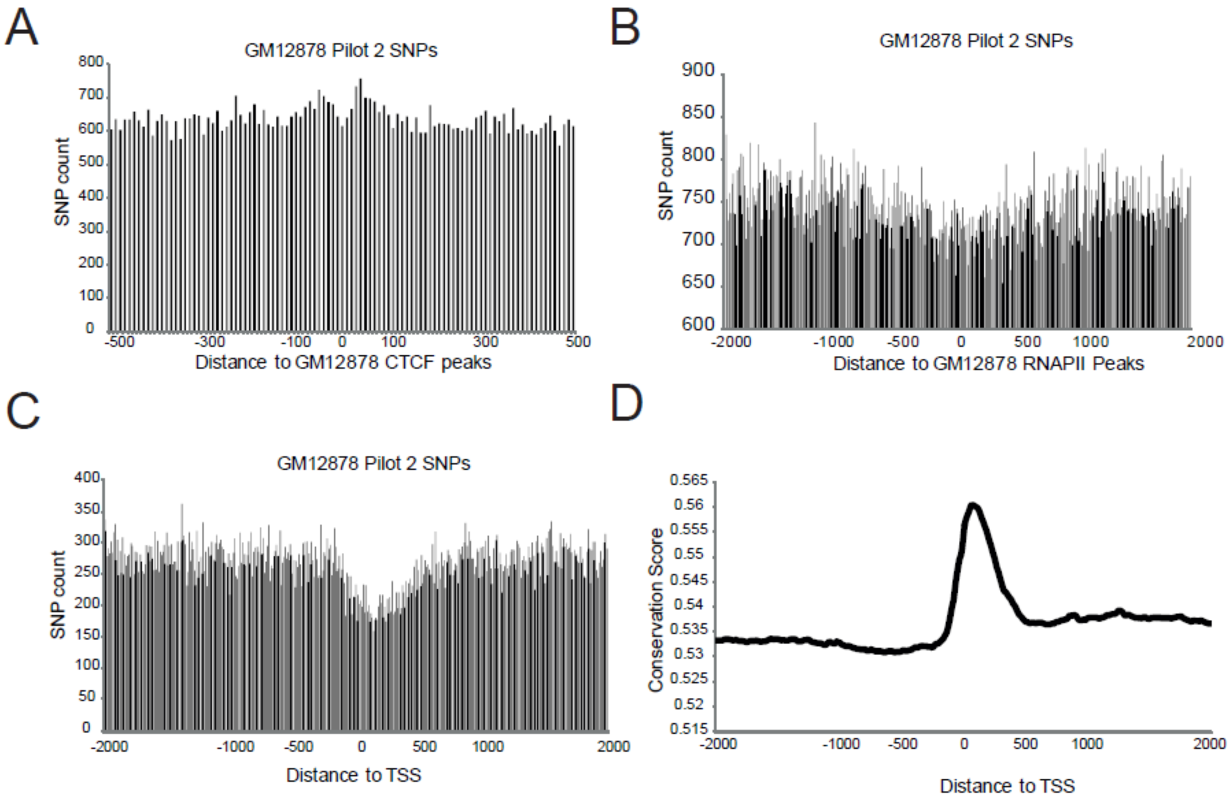

Supplemental Figure S6. Pilot 2 SNP distribution around (A) CTCF and (B) RNAPII ChIP peak centers and (C) transcription start sites. (D) Conservation scores around transcription start sites. All distances are in bp.

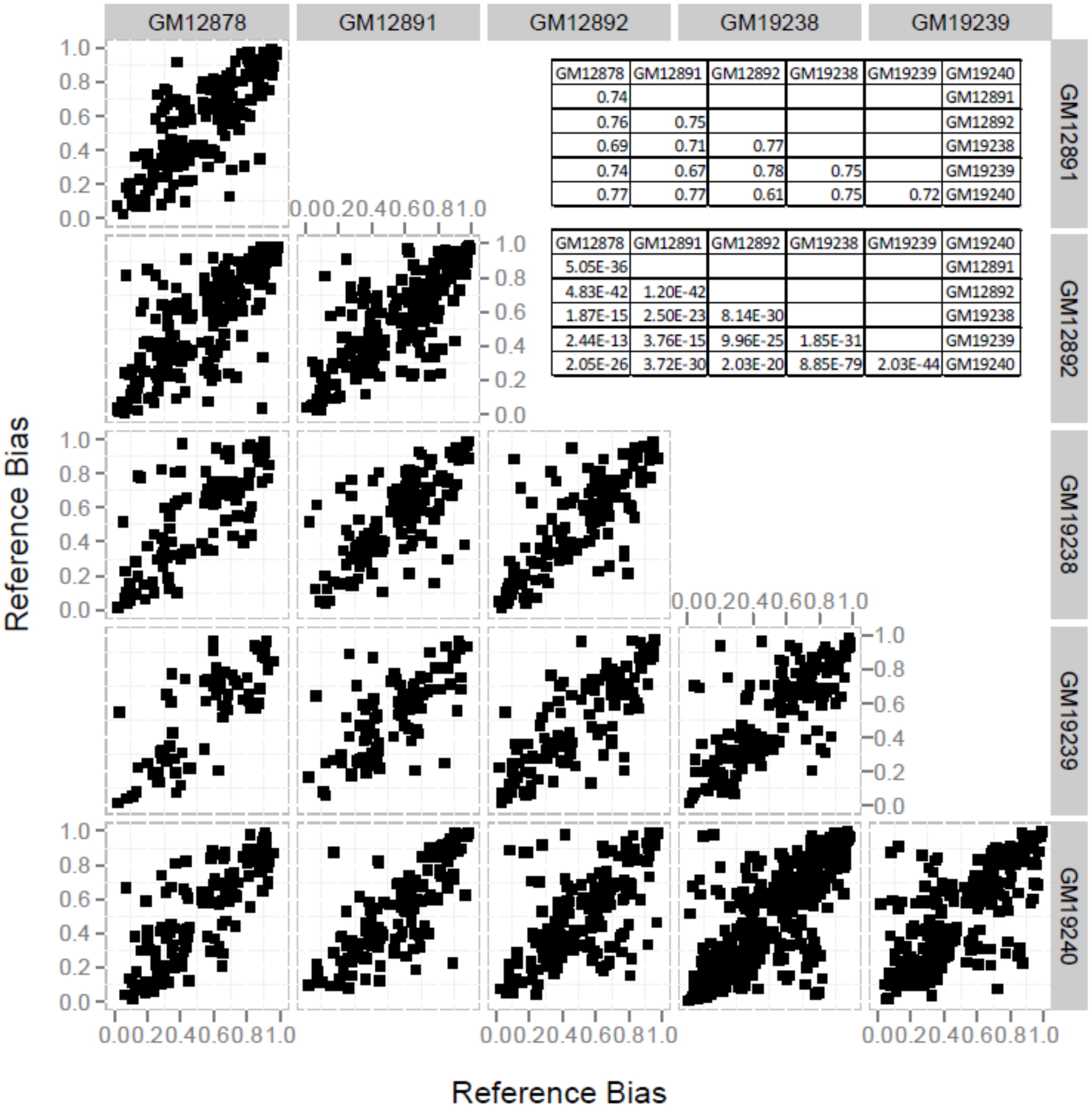

GM12878	GM12891	GM12892	GM19238	GM19239	GM19240
0.74					GM12891
0.76	0.75				GM12892
0.69	0.71	0.77			GM19238
0.74	0.67	0.78	0.75		GM19239
0.77	0.77	0.61	0.75	0.72	GM19240

GM12878	GM12891	GM12892	GM19238	GM19239	GM19240
5.05E-36					GM12891
4.83E-42	1.20E-42				GM12892
1.87E-15	2.50E-23	8.14E-30			GM19238
2.44E-13	3.76E-15	9.96E-25	1.85E-31		GM19239
2.05E-26	3.72E-30	2.03E-20	8.85E-79	2.03E-44	GM19240

Supplemental Figure S7. CTCF allelic binding bias at Pilot 2 SNPs was plotted similarly as in Fig. 5. The inset tables show the Spearman correlation coefficients (top) and Spearman *P* values (bottom).

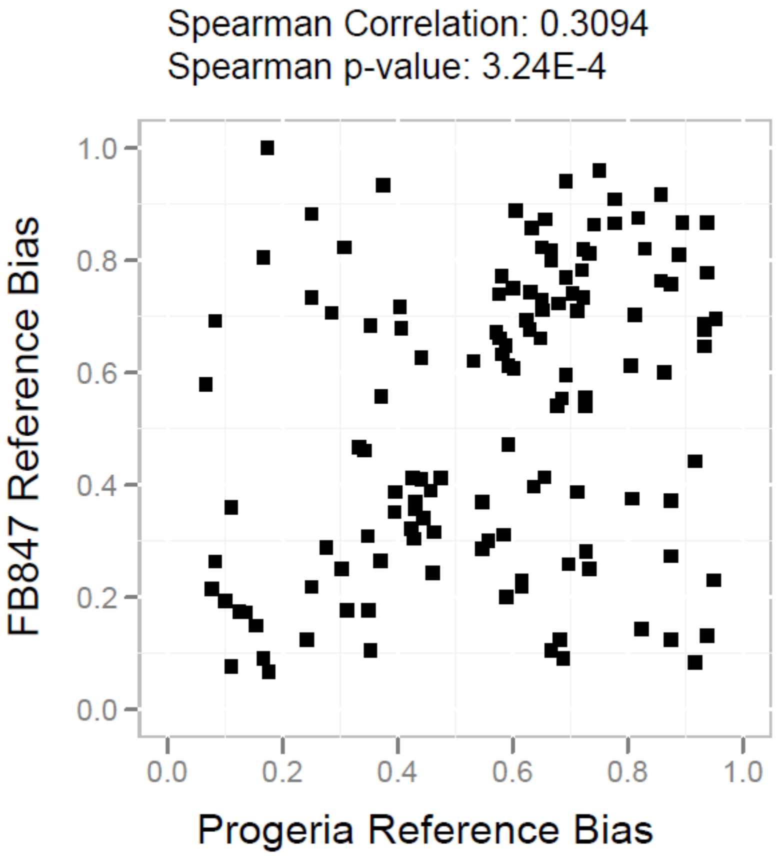

Supplemental Figure S8. CTCF allelic binding bias at discovered SNPs in Progeria and FB8470 (normal) fibroblast cells.

Error	Cell line/ Factor	Coordinate	Genotype call from ChIP-seq	Genotype from Sanger sequencing of genomic DNA	Known alleles from dbSNP 129 (ref/alt)	Genotype from 1000 Genomes Pilot 2
1	GM12878/ CTCF	chr8:27777665	G/G	C/G	C/G	C/G
2	GM12878/ CTCF	chr9:1722880	G/A	A/A	G/A	G/A
3	GM12878/ CTCF	chr2:11212584	A/A	G/A	G/A	Not called
4	GM12878/ RNAPII	chr6:26139592	G/A	A/A	G/A	G/A
5	Progeria/ RNAPII	chr14:68323944	C/T	T/T	C/T	Not available
6	Progeria/ RNAPII	chr6:32509195	C/T	C/C	C/T	Not available

Supplemental Table S1. Description of apparent errors. This table lists all 6 discrepancies that we observed between genotypes called from ChIP-seq data and our genomic Sanger sequencing validation (127 out of 133 were exactly correct). For errors 1 and 3, the ChIP-seq data recovered the alternate allele and called it homozygous, but the reference allele was apparently not observed at sufficient coverage. Errors 2 and 4 are discrepant between the ChIP-seq and Sanger genotyping, but our ChIP-seq call matched the 1000 Genomes Pilot 2 genotype. For errors 5 and 6, the ChIP-seq data called it heterozygous and Sanger sequencing reported homozygous (similar to errors 2 and 4), but the two alleles reported by ChIP-seq correspond to the two alleles known to occur at that position (in other individuals) according to dbSNP 129.

Cell	GM12878	GM12891	GM12892	GM19238	GM19239	GM19240	GM12878	GM12878	GM12878
Factor	CTCF	CTCF	CTCF	CTCF	CTCF	CTCF	RNAPII	H3K4me3	H3K27me3
ChIP-seq indels	806	777	1173	1153	1068	985	525	4031	464
1000 Genomes Project indels	328527	326650	311056	361678	383696	402234	328527	328527	328527
Overlap	519	473	697	781	672	707	292	2396	249
Percent overlap	64.39%	60.88%	59.42%	67.74%	62.92%	71.78%	55.62%	59.44%	53.66%

Supplemental Table S2. Indels called from ChIP-seq data overlap with 1000 Genomes Project indel calls.

Cell	GM12878	GM12891	GM12892	GM19238	GM19239	GM19240	GM12878	GM12878
Factor	CTCF	CTCF	CTCF	CTCF	CTCF	CTCF	Pol2	H3K4me3
Novel SNPs	6622	13165	16023	11958	8678	7949	9955	13675
Novel SNPs found in corresponding CEU or YRI low coverage populations	5579	11377	13597	9315	6456	5664	8107	10953
Percent	84.25%	86.42%	84.86%	77.90%	74.40%	71.25%	81.44%	80.10%

Supplemental Table S3. Novel SNPs found by ChIP-seq overlap with SNPs found in other individuals in the same population in the 1000 Genomes Project low coverage data.

	GM12878	GM12891	GM12892	GM19238	GM19239	GM19240
ChIP-seq biased SNPs	133	705	394	364	120	427
Pilot 2 biased SNPs	375	680	757	774	377	961
Overlap	117	328	308	289	99	376

Supplemental Table S4. Overlap between biased (that is, allele-specific) SNPs discovered from ChIP-seq data and biased Pilot 2 SNPs.

GWAS SNP					ChIP-seq site SNP		
**chr**	**pos**	**dbSNP**	**trait(s)**	**SNP class**	**cell**	**pos**	***P*-val**
1	23409478	rs1738475	Height	Intergenic	FB8470	23409478	8.8E-03
	59571749	rs17119280	Response to antipsychotic therapy (extrapyramidal side effects)	intron	FB8470	59572099	2.8E-02
	62769426	rs1168013	Triglycerides	intron	FB8470	62768961	3.7E-10
	203980001	rs823128	Parkinson's disease	intron	FB8470	203980422	3.9E-02
	242240495	rs10927101	Diabetic retinopathy	Intergenic	GM12892	242240273	1.9E-03
2	216606903	rs7590720	Alcohol dependence	Intergenic	FB8470	216607365	8.8E-03
3	197293536	rs11915082	Mean corpuscular hemoglobin	nearGene-5	GM19240	197293513	4.1E-02
					GM12891	197293536	2.3E-02
6	29778240	rs3129055	Nasopharyngeal carcinoma	Intergenic	GM12892	29778377	1.1E-02
					GM19238	29778377	2.3E-02
					GM19240	29778377	4.7E-02
	32331236	rs3130320	Systemic lupus erythematosus	Intergenic	Progeria	32331000	2.6E-02
	32698903	rs3129763	Systemic sclerosis	Intergenic	FB8470	32698451	2.5E-05
					GM12891	32698451	3.3E-04
9	111985226	rs7032940	Height	Intergenic	FB8470	111985595	3.9E-02
10	30356078	rs3739998	Coronary heart disease	missense	GM12892	30356214	3.8E-02
12	51560171	rs902774	Prostate cancer	Intergenic	GM12892	51560171	2.7E-02
15	87703036	rs4932217	Height	null	GM12891	87703386	1.6E-02
					Progeria	87703386	1.1E-02
16	55562980	rs1532624	Cholesterol,HDL cholesterol	intron	GM12891	55562802	4.0E-03
	80935282	rs4087296	Bone mineral density	Intergenic	Progeria	80935427	4.4E-02
					FB8470	80935545	5.9E-03
					Progeria	80935545	4.4E-02
18	54903034	rs1037757	Alzheimer's disease (age of onset)	null	GM12891	54902626	2.5E-02
20	3724175	rs3761218	Bipolar disorder	nearGene-5	GM12891	3724368	1.7E-02
21	41667951	rs45430	Melanoma	intron	GM19240	41668146	2.2E-02
22	40548802	rs7364180	Alzheimer's disease biomarkers	intron	Progeria	40548802	3.2E-02

Supplemental Table S5. Significantly biased allele-specific CTCF binding sites within 500 bp of a GWAS SNP locus. *P*-val refers to the significance of the allele-specificity binding bias at a heterozygous SNP.

Cell line	factor	aligned reads	total SNPs	Ti/Tv
BJ	H3K27me3	30,273,340	151,340	2.06
Caco-2	H3K27me3	34,819,190	170,557	2.10
H1 ESC	H3K27me3	20,220,420	99,263	2.06
GM06990	H3K27me3	30,264,409	139,719	2.12
GM12878	H3K27me3	51,982,091	298,640	2.06
HelaS3	H3K27me3	30,345,413	43,931	2.03
HepG2	H3K27me3	23,197,669	74,222	2.11
HMEC	H3K27me3	20,434,567	43,256	2.03
HRE	H3K27me3	36,192,891	215,694	2.11
HSMM	H3K27me3	23,379,041	68,443	2.05
HUVEC	H3K27me3	57,397,946	275,756	2.10
K562	H3K27me3	46,640,156	205,480	2.09
NHEK	H3K27me3	50,515,220	149,113	2.05
NHLF	H3K27me3	19,774,591	53,873	2.09
SAEC	H3K27me3	32,150,308	180,309	2.06
SK-N-SH_RA	H3K27me3	31,594,484	160,784	2.06
BJ	H3K4me3	31,152,288	67,280	1.97
Caco-2	H3K4me3	29,646,596	74,480	2.04
H1 ESC	H3K4me3	21,358,289	110,286	1.97
GM06990	H3K4me3	32,937,237	82,018	2.05
H160	H3K4me3	22,692,790	46,100	2.03
HelaS3	H3K4me3	30,366,999	105,902	2.03
HepG2	H3K4me3	50,408,596	124,436	2.05
HMEC	H3K4me3	29,985,385	102,069	2.01
HRE	H3K4me3	30,025,651	63,870	2.00
HSMM	H3K4me3	21,635,033	66,901	2.06
HUVEC	H3K4me3	56,647,267	161,221	2.04
K562	H3K4me3	24,072,731	126,260	2.03
NHEK	H3K4me3	45,280,559	122,845	2.02
NHLF	H3K4me3	34,150,595	156,132	2.02
SAEC	H3K4me3	29,422,553	69,087	1.99
SK-N-SH_RA	H3K4me3	31,981,702	53,418	1.94
GM12891	RNA-seq	42,577,447	42,101	2.38

Supplemental Table S6. SNP calling from H3K4me3 and/or H3K27me3 ChIP-seq data in 17 additional cell lines (ENCODE data) as well as from RNA-seq data in GM12891 (from Toung *et al*., Genome Res. (2011) 21:991-8)
